# Cytotoxic T lymphocyte-associated antigen-4-Ig (CTLA-4-Ig) suppresses *Staphylococcus aureus*-induced CD80, CD86, and pro-inflammatory cytokine expression in human B cells

**DOI:** 10.1186/s13075-020-2138-x

**Published:** 2020-03-30

**Authors:** Po-Chun Liu, Chih-Tai Ssu, Yen-Po Tsao, Teh-Ling Liou, Chang-Youh Tsai, Chung-Tei Chou, Ming-Han Chen, Chuen-Miin Leu

**Affiliations:** 1 Institute of Microbiology & Immunology, National Yang-Ming University, 155 Sec. 2, Li-Nong St., Taipei City, 11221 Taiwan; 2 Division of Allergy, Immunology & Rheumatology, Department of Medicine, Taipei Veterans General Hospital, No. 201, Sec. 2, Shih-Pai Road, Taipei City, 11217 Taiwan; 3 Faculty of Medicine, National Yang-Ming University, Taipei City, Taiwan; 4 Infection and Immunity Center, National Yang-Ming University, Taipei City, Taiwan, Republic of China

**Keywords:** CTLA-4, CD80, CD86, TNF-α, IL-6, Rheumatoid arthritis, Abatacept

## Abstract

**Background:**

Cytotoxic T lymphocyte-associated antigen-4-Ig (CTLA-4-Ig) competes with CD28 for binding CD80/CD86 on antigen-presenting cells (APCs) to limit T cell activation. B cells are believed to be important APCs in the pathogenesis of autoimmune diseases and express CD80/CD86 after activation; however, relatively little is known about the effect of CTLA-4-Ig on B cells. This study tested the impact of CTLA-4-Ig on human B cell responses.

**Methods:**

Human blood B cells were purified from healthy donors and activated in the presence of CTLA-4-Ig or the L6-Ig control protein in vitro. RT-q-PCR and immunofluorescence staining were performed to detect activation marker expression. ELISA was conducted to measure cytokine secretion. The CD80/CD86 levels on the surface of the memory B cells in the blood of 18 patients with rheumatoid arthritis (RA) were detected using immunofluorescence staining.

**Results:**

CTLA-4-Ig suppressed the expression of *Staphylococcus aureus* (SAC)-induced *CD80*, *CD86*, *TNFA*, and *IL6* in human B cells at the transcriptional level. Furthermore, CTLA-4-Ig concomitantly decreased SAC-induced CD80/CD86 surface expression on and TNF-α and IL-6 secretion from B cells. On the other hand, T cell-dependent (TD) stimulation-induced B cell activation, proliferation, plasma cell differentiation, and antibody secretion were not affected by CTLA-4-Ig. As expected, TD stimulation-induced surface CD80 was hindered by CTLA-4-Ig. Notably, a blockade of CD80/CD86 on the surface of the memory B cells was observed in the patients with RA after abatacept (CTLA-4-Ig) treatment. In a portion of the RA patients, restoration of CD80/CD86 staining on the surface of the memory B was detected starting in the 3rd month of abatacept treatment. Interestingly, the surface levels of CD80/CD86 on the patients’ memory B cells positively correlated with disease activity.

**Conclusions:**

We found that CTLA-4-Ig directly suppressed SAC-induced B cell activation in vitro. Obstruction of CD80 and CD86 on the surface of the memory B cells was detected in the RA patients after abatacept treatment. Blocking CD80/CD86 on B cells by CTLA-4-Ig may hinder T cell activation and associated with the disease activity of RA in vivo. Our findings indicate that CTLA-4-Ig may regulate humoral responses by modulating B cell activation and interfering T cell-B cell interaction.

## Background

Cytotoxic T lymphocyte-associated protein 4 (CTLA-4) is a negative costimulatory molecule expressed on the surface of activated and regulatory T cells. Deletion of CTLA-4 in mice causes autoimmune disease symptoms, underlining the importance of CTLA-4 in maintaining self-tolerance and immune homeostasis [1–3]. CTLA-4 effectively attenuates T cell activation via competing with the costimulatory molecule CD28 to bind with ligands CD80 (B7.1) and CD86 (B7.2) on antigen-presenting cells (APCs), for which it has a higher affinity and avidity than does CD28 [4]. Because of the potent inhibition of CTLA-4, a fusion protein composed of the extracellular domain of CTLA-4 and the IgG1 Fc portion (CTLA-4-Ig) was developed and approved to alleviate unwanted immune responses in autoimmune diseases. For instance, abatacept (CTLA-4-Ig) is effective in alleviating disease activity in rheumatoid arthritis (RA) patients who have an inadequate response to methotrexate or anti-tumor necrosis factor (TNF)-α therapies [5–8].

RA is the most common form of chronic inflammatory arthritis and its etiology remains unknown. Both innate and adaptive immune systems participate in the pathogenesis of RA. In addition to T cells and myeloid cells, B cells play a crucial role in the pathogenesis of RA, as indicated by the observation that depletion of B cells is very effective for ameliorating RA [9, 10]. Autoreactive B cells contribute to inflammation by producing autoantibodies, proinflammatory cytokines, and chemokines [11, 12]. Moreover, B cells are the most abundant and important APCs that interact with T cells in autoimmune diseases, specifically, activating and expanding effector and memory T cells [13–15].

Mechanistically, CTLA-4 regulates immune responses by competing with CD28 for CD80/CD86 binding, transducing an inhibitory signal in T cells, and shortening the time of contact between APCs and T cells [4]. Similar to its membrane form, soluble CTLA-4-Ig acts by competing with CD28 for CD80/CD86 binding and modulates the costimulatory signaling necessary for full T cell activation [16]. CTLA-4-Ig treatment in vivo suppressed T cell-dependent (TD) and T cell-independent (TI) antibody responses in humans and mice [17–19]. In vitro, CTLA-4-Ig binds dendritic cells and induces the activation of the tryptophan-degrading enzyme indoleamine 2,3-dioxygenase (IDO), which depletes the essential amino acid tryptophan and leads to suppression of T cell activation or induces the secretion of the immunosuppressive HLA-G molecule [20, 21]. CTLA-4-Ig is also able to bind synovial macrophages from RA patients and suppress IL-6 and TNF-α production [22]. These observations reveal the ability of CTLA-4-Ig to induce signaling to modulate cytokine production in dendritic cells and macrophages.

CTLA-4-Ig has been reported to reduce B cell infiltrates in synovial biopsy samples [23] and suppress humoral responses against TD and TI antigens in vivo [17–19]. Since T cells are critical for the activation and differentiation of B cells, whether CTLA-4-Ig directly modulates B cell functions has not been effectively elucidated. Using an in vitro system, a recent study discovered that abatacept directly decreased TD stimulation-induced CD80/CD86 expression via dynamin-dependent internalization [24]. Other reports demonstrated that anti-CD86 monoclonal antibodies (mAbs) enhanced LPS-induced proliferation and IgG1 and IgG2a production [25] and increased TD Ag-stimulated IgG1 and IgE production in mouse B cells in vitro [26–28]. In human tonsillar B cells, anti-CD86 mAbs caused a modest increase in IgE and IgG4 [29]. Collectively, these results suggest that CD80 and CD86 are able to transduce a signal that regulates antibody production by mouse and human B cells, and CTLA-4-Ig binding to human B cells resulted in a reduction in surface CD80 and CD86. Whether CTLA-4-Ig binding to human B cells directly modulates activation-induced gene expression, proliferation, and antibody secretion is not clear.

Given that B cells express CD80 and CD86 after activation, we hypothesize that CTLA-4-Ig binding to B cells may regulate B cell responses. To test this hypothesis, we stimulated purified human B cells from healthy donors in the presence of CTLA-4-Ig and evaluated B cell activation level, proliferation, plasma cell differentiation, and antibody production in vitro. Furthermore, we studied the in vivo effect that abatacept has on the levels of CD80/CD86 on the surface of the memory B cells in the blood of the RA patients. The correlation between CD80/CD86 levels and disease activity in RA patients was analyzed.

## Methods

### Human subjects

Eighteen patients with RA and 24 healthy controls were enrolled in the study. The diagnosis of RA was based on the 2010 American College of Rheumatology/European League Against Rheumatism collaborative initiative (ACR/EULAR) classification criteria [30]. Abatacept (CTLA-4-Ig) was prescribed to the RA patients who were refractory to methotrexate. Clinical and laboratory assays of RA were recorded. RA disease activity was measured using Disease Activity Score 28 with ESR (DAS28-ESR) [31]. Table 1 shows the demographic, laboratory, and clinical characteristics of the 18 patients with RA refractory to methotrexate. Among them, 10 (55.6%) were biologically naïve, while 8 (44.4%) had a history of one or more previous biologic therapies. Over 80% of the patients were seropositive for rheumatoid factor (RF), and approximately 60% were positive for anti-cyclic citrullinated peptide antibody (ACPA). In the recruited RA patients, the mean baseline erythrocyte sedimentation rate (ESR) and DAS28-ESR were 41.1 mm/h and 5.3, respectively. This study was approved by the institutional ethics committees of Taipei Veterans General Hospital and National Yang-Ming University, and informed consent was signed by all patients and healthy donors before they participated in this study. All samples were collected in compliance with the Declaration of Helsinki.

**Table 1:** Baseline characteristics of the enrolled RA patients

	*N* = 18
Age of diagnosis, years (mean ± SD)	52.6 ± 10.9
Female, *n* (%)	17 (94.4)
Disease duration prior to abatacept, years, median (range)	5.8 (0.8~30.0)
Number of previous biologics	
0, *n* (%)	10 (55.6)
1, *n* (%)	3 (16.7)
2, *n* (%)	3 (16.7)
3, *n* (%)	2 (11.1)
Rheumatoid factor positive, *n* (%)	16 (88.9)
Anti-CCP antibody positive, *n* (%)	11 (61.1)
ESR, mm/h (mean ± SD)	41.1 ± 22.7
CRP, mg/dl, median (range)	1.0 (0.3~7.7)
Tender joint count (mean ± SD)	4.3 ± 3.2
Swollen joint count, median (range)	5 (1~12)
VAS ptGA, mm (from 0 to 100) (mean ± SD)	68.8 ± 21.9
DAS28-ESR score (mean ± SD)	5.3 ± 1.2

Normally distributed data are represented as mean ± standard deviation (SD). *Abbreviations*: *Anti-CCP antibody* anti-cyclic citrullinated peptide antibody, *ESR* erythrocyte sedimentation rate, *CRP* C-reactive protein, *VAS ptGA* patient global assessment on a visual analog scale, *DAS28-ESR* Disease Activity Score 28 using ESR

### EULAR response criteria

Clinical responses to abatacept therapy were assessed using the EULAR criteria [32]. Patients were classified as good responders, moderate responders, and nonresponders according to DAS28-ESR levels and their improvement with respect to the baseline. Briefly, a good EULAR response was defined as DAS28-ESR change > 1.2 with DAS28-ESR ≤ 3.2, a moderate EULAR response was defined as DAS28-ESR change > 0.6 with DAS28-ESR > 3.2–5.1, while a nonresponse was defined as DAS28-ESR change ≤ 0.6 and absolute DAS28-ESR > 5.1. The clinical response status was evaluated 6 months after abatacept treatment.

### Human B cell purification

Blood was obtained in accordance with the policies established by the National Yang-Ming University Institutional Review Board. Blood was withdrawn from healthy donors, and peripheral blood mononuclear cells (PBMCs) were isolated using Ficoll-Paque PLUS gradient centrifugation (GE Healthcare, Chicago, IL). B cells were positively isolated from PBMCs using CD19 microbeads (Miltenyi Biotec, Auburn, CA). Cell purity was determined using anti-human CD20-APC (BioLegend, San Diego, CA) staining. B cell purity using CD19 microbeads purification was > 95% in all experiments. Cells were cultured in RPMI 1640 medium containing 100 U/ml penicillin, 100 mg/ml streptomycin, 2 mM L-glutamine, and 10% fetal calf serum (Life Technologies, Grand Island, NY). CTLA-4-Ig and L6-Ig (control-Ig) for use in the in vitro assays were provided by Bristol-Myers Squibb. L6-Ig is a chimeric fusion protein consisting of the V region of the murine L6 antigen and the human IgG1 Fc portion, and it was used as a control in our studies.

### [^3^H]-thymidine incorporation assays

Fifty thousand purified human B cells were seeded in a 96-well plate and treated with the indicated reagents for 3 days. Then, 1 μCi [^3^H]-thymidine was added to each well and incubated for an additional 24 h. The cells were harvested, and ^3^H-thymidine incorporation was assessed with a liquid scintillation counter (Perkin Elmer). Anti-CD40 antibody (5 μg/ml, R&D Systems, Minneapolis, MN), anti-IgM antibody (1 μg/ml, Jackson ImmunoResearch Inc., West Grove, PA), SAC (formalin-fixed *Staphylococcus aureus*, Cowan I strain, 1:10000 dilution, MERCK), CTLA-4-Ig, and L6-Ig control protein were also used in the study.

### Fluorescent staining and flow cytometry analysis

For fluorescent staining, 10^6^ cells were washed with ice-cold PBS and then blocked with Human TruStain-fcX (BioLegend) for 15 mins on ice. Specific antibodies for staining were added and incubated for 15 mins on ice, and then, the cells were washed with 1 ml ice-cold FACS buffer 3 times. Anti-CD27-FITC, anti-CD69-PE, anti-CD80-PE, and anti-CD86-PE were purchased from BioLegend. For a biotinylated CTLA-4-Ig binding assay, streptavidin-PE was added after the cells were washed. In some experiments, 400 μl of 2% formalin in PBS was used to fix the cells before analysis using flow cytometry (BD Biosciences FACSCanto). Data were analyzed by using Flow Jo software. Dead cells were excluded using propidium iodide, and doublet discrimination was determined by plotting FSC-H vs. FSC-A.

### Acidic elution of surface bound CTLA-4-Ig

Purified blood CD19^+^ B cells were stimulated with SAC or anti-IgM plus anti-CD40 antibodies in the presence of 100 μg/ml CTLA-4-Ig or L6-Ig (control-Ig) for 2 days. The treated cells were harvested and washed twice with ice-cold PBS. To remove surface bound CTLA-4-Ig or control-Ig, cell pellets were resuspended in acid elution buffer (133 mM citric acid and 66 mM Na_2_HPO_4_, pH 3.0) for 4 min at room temperature. The cells were washed twice with ice-cold PBS containing 2% heat-inactivated normal goat serum before being stained with the F(ab′)_2_ portion of the goat anti-human IgG-Fc antibody (Jackson ImmunoResearch) or anti-CD80/CD86 antibody. Fluorescent staining and flow cytometry analysis were conducted as described above.

### Measurement of cytokine production and secretion

Two hundred thousand purified B cells were seeded in a 96-well plate and stimulated with the indicated reagents in the presence of CTLA-4-Ig or the L6-Ig control protein. The supernatant and the cells were collected either at 8 h (for detection of TNF-α) or at 48 h (for IL-6 and other genes) after stimulation, and ELISA or RT-q-PCR were used to quantify the levels of CD80, CD86, and other cytokines. After RNA was converted to cDNA, Maxima SYBR Green qPCR mix (Thermo Scientific) was used to conduct q-PCR. Human TNF-α and IL-6 were detected using a human cytokine ELISA Set (RUO) purchased from BD Biosciences.

### ELISPOT assay

The ELISPOT assay was performed using a human IgG B cell ELISpot development module (R&D Systems GmbH), and all procedures were performed according to the manufacturer’s instructions. In brief, purified human B cells were stimulated in complete RPMI 1640 with 5 μg/ml anti-IgM, 1 μg/ml anti-CD40, and 100 ng/ml human IL-21 (R&D Systems GmbH). After 4 days, the cells were washed, and 2000 cells were seeded in each well in quintuplicate in a MultiScreen HTS IP filter plate (Millipore) precoated with goat anti-human IgG polyclonal antibody (R&D Systems GmbH). After 16 h, the cells were washed off, and the plate was incubated overnight with biotinylated goat anti-human IgG polyclonal antibody (R&D Systems GmbH) at 4 °C. ELISPOTs were then developed by an ELISpot blue color module (R&D Systems GmbH). The spot number and size were measured by AID EliSpot/FluoroSpot reader systems (Autoimmun Diagnostika GmbH).

### Statistical analysis

The results were analyzed using the Mann-Whitney *U* test and expressed as the mean ± standard deviation (SD). The differences were considered to be significant when *p* < 0.05, *p < 0.05, **p < 0.01, or ***p < 0.005.

## Results

### CTLA-4-Ig binds human peripheral blood memory and activated B cells

**Fig. 1:**
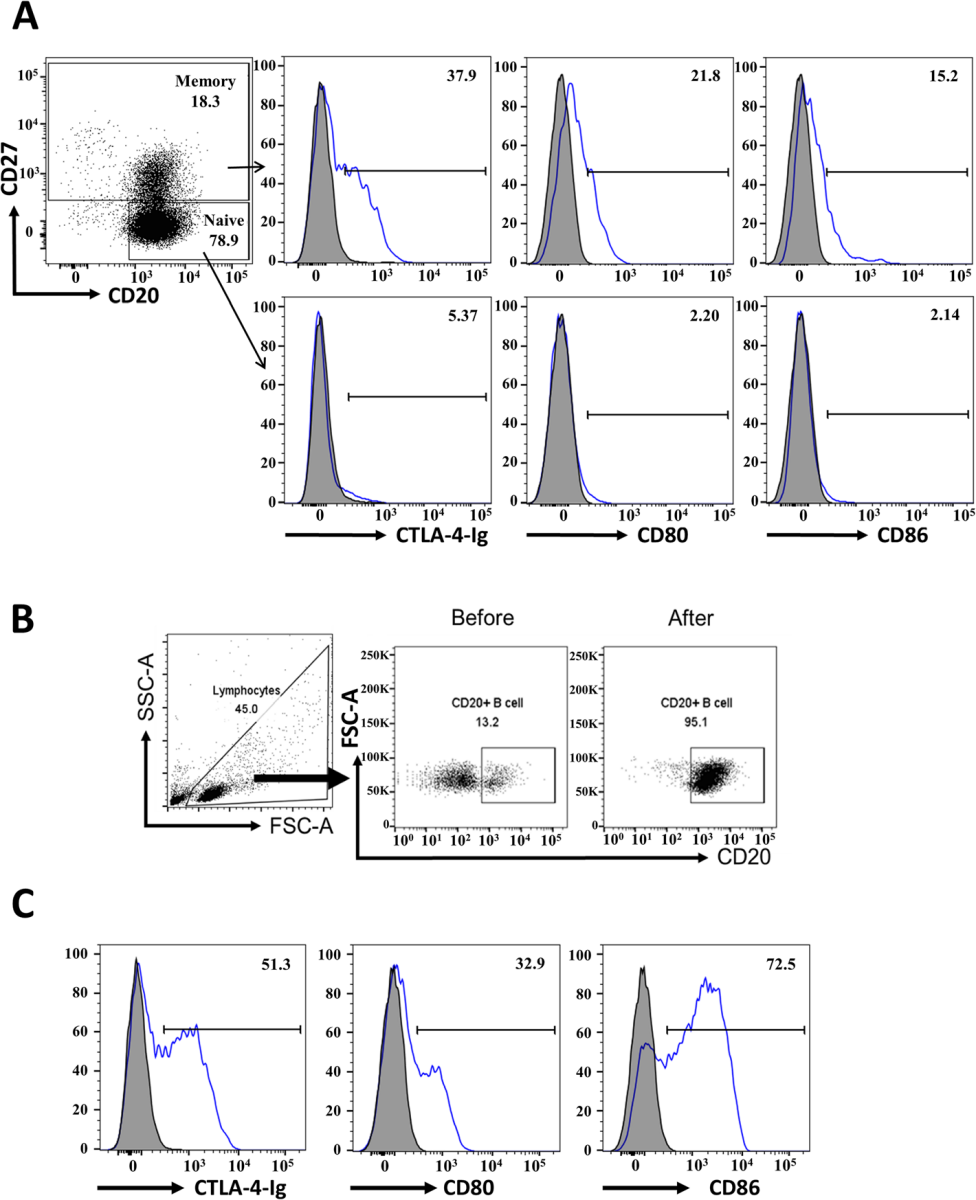
CTLA-4-Ig binds human memory and activated B cells. **a** CTLA-4-Ig binds peripheral blood memory B cells. Human peripheral blood mononuclear cells (PBMCs) were stained with 10 μg/ml biotinylated-CTLA-4-Ig or control-protein (Ctrl-Ig), followed by streptavidin-PE together with anti-CD20 and anti-CD27 antibodies and analyzed using flow cytometry. Left histograms, one representative binding of CTLA-4-Ig gated on CD20^+^CD27^−^ naïve B cells (bottom) or on CD20^+^CD27^+^ memory B cells (top) is shown (*n* = 3). Gray peaks, Ctrl-Ig; blue line, CTLA-4-Ig. Middle and right histograms, anti-CD80 or anti-CD86 antibody was used to examine CD80 and CD86 expression on naïve and memory B cells. Gray peak, isotype control; blue line, anti-CD80 or anti-CD86 antibody. **b** CD19^+^ B cells were purified from the blood of healthy donors and the purity was determined by anti-CD20 antibody staining. On representative result is shown. **c** CTLA-4-Ig binds activated B cells. Purified CD19^+^ B cells from blood were stimulated with anti-IgM and anti-CD40 antibodies for 3 days and the levels of CTLA-4-Ig binding (left), CD80 (middle), and CD86 (right) were examined as described in **a**. Gray peaks, Ctrl-Ig (10 μg/ml) or isotype control; blue line, CTLA-4-Ig (10 μg/ml) or anti-CD80/86 antibody. Data are representative of three independent experiments

To test the possible effect of CTLA-4-Ig on human B cells, we first examined whether it binds B cells. When peripheral blood B cells were incubated with biotinylated CTLA-4-Ig or control-Ig protein with streptavidin-PE subsequently added, we found that CTLA-4-Ig bound ~ 38% of the memory B (CD20^+^CD27^+^) but not the naïve B cells (CD20^+^CD27^−^) (Fig. 1a). The binding of CTLA-4-Ig correlated with the CD80 and CD86 levels on the surface of the B cells, as inferred since memory B cells express higher levels of CD80 and CD86 than are expressed by naïve B cells (Fig. 1a); this finding is consistent with a published report [33]. Because B cells upregulate CD80 and CD86 expression after activation, we tested whether CTLA-4-Ig would have increased its binding on activated human B cells. CD19^+^ B cells were purified, and the purity was over 95% for all the experiments in this study (Fig. 1b). When blood B cells were stimulated with anti-IgM and anti-CD40 antibodies, they exhibited increased surface levels of CD80 and CD86 (Fig. 1c and supplemental Fig. 1A). Under these conditions, CTLA-4-Ig showed dose-dependent binding to the activated B cells (supplemental Fig. 1B). At a dose of 1 μg/ml, CTLA-4-Ig bound 24% of the B cells, a percentage comparable to that of the CD80^+^ B cells. At 10 μg/ml, more than 50% of the B cells bound CTLA-4-Ig (Fig. 1c and supplemental Fig. 1B). At 100 μg/ml, 85% of the B cells bound CTLA-4-Ig, a percentage similar to that of the CD86^+^ B cells (supplemental Fig. 1B). These results indicate that, at lower concentrations, only CD80 is bound by CTLA-4-Ig. At increased concentrations, CTLA-4-Ig binds both CD80 and CD86 on activated B cells. On the basis of these observations, we found that CTLA-4-Ig binds memory and activated B cells, suggesting its potential to influence B cell functions.

### Effect of CTLA-4-Ig on SAC- or T cell-dependent stimulation-induced B cell activation

**Fig. 2:**
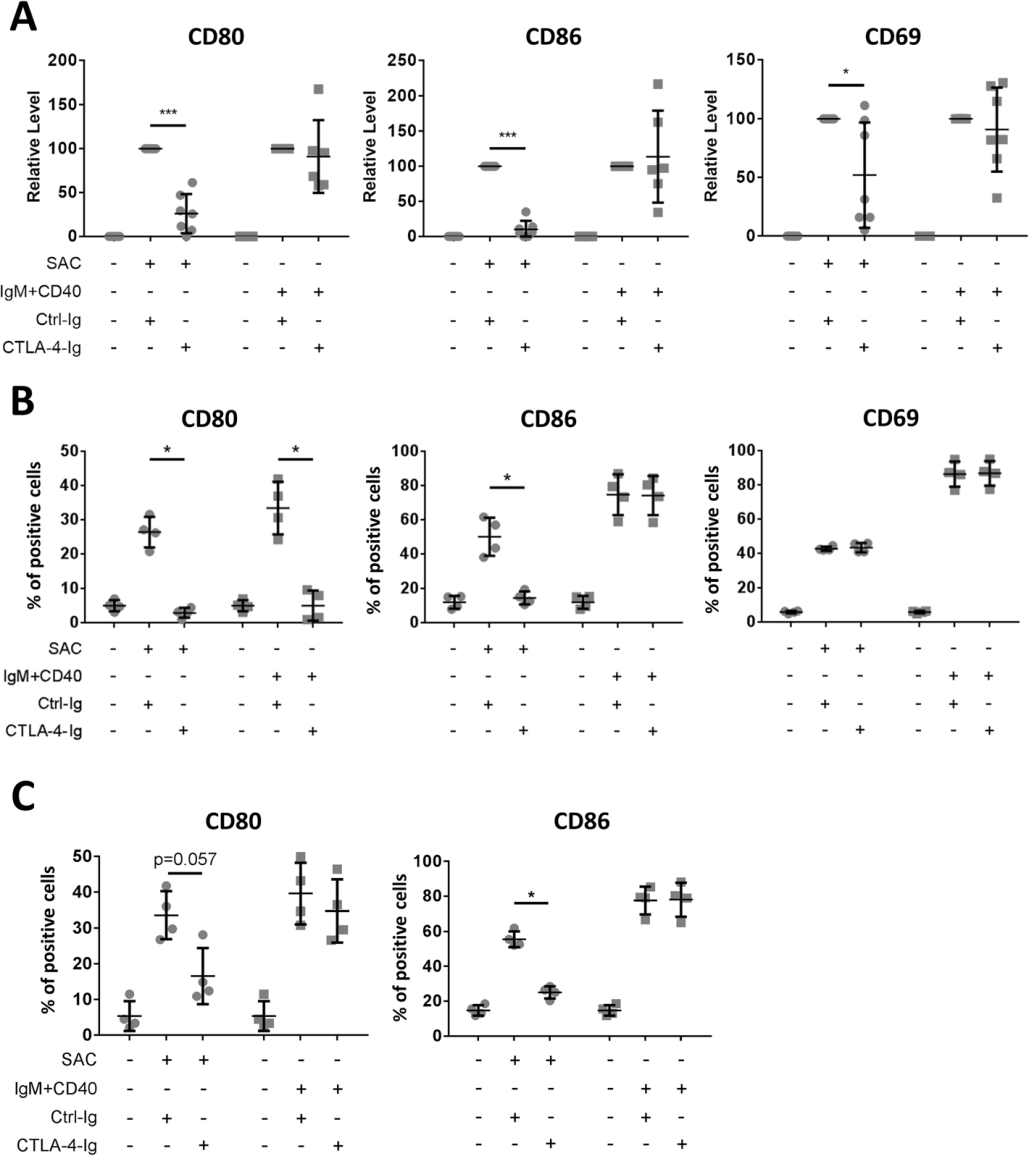
CTLA-4-Ig suppresses *Staphylococcus aureus*-induced CD80 and CD86 levels on B cells in vitro. **a** Purified CD19^+^ B cells were stimulated with SAC (*Staphylococcus aureus* Cowan strain, SAC) or anti-IgM (5 μg/ml) plus anti-CD40 (1 μg/ml) antibodies in the presence of CTLA-4-Ig (100 μg/ml) or Ctrl-Ig (100 μg/ml) for 2 days. The expression of CD80, CD86, and CD69 was measured by RT-q-PCR, and GAPDH level was used to normalize the readout. After deducing the value of the medium control, the value of the control-Ig-treated B cells obtained from each donor was considered to be 100% for calculation of the relative level. Each dot represents one donor (*n* = 7~8). **b** The effect of CTLA-4-Ig on the level of CD80, CD86, or CD69 on the surface of the B cells was analyzed using flow cytometry (*n* = 4). The percentage of positive cells in each experiment is shown. **c** CD19^+^ B cells were stimulated as described in **a**. After acidic elution to remove bound CTLA-4-Ig, the CD80 and CD86 on the surface of the treated cells were examined using immunofluorescence staining. The percentage of positive cells in each experiment is shown (*n* = 4)

To test whether CTLA-4-Ig regulates B cell activation, the *Staphylococcus aureus* Cowan (SAC) strain was used to induce TI activation, and anti-IgM plus anti-CD40 antibodies were used to mimic TD activation in PBMC B cells. We first chose a near saturation concentration of CTLA-4-Ig (100 μg/ml) to examine its possible effect. Using RT-q-PCR to detect three activation markers, we found that the SAC-induced *CD80* and *CD86* expression in the human B cells was significantly decreased by CTLA-4-Ig but not by the control protein (Fig. 2a). CTLA-4-Ig reduced *CD80* expression in 7 of 8 donors with an average inhibition of 74.0 ± 22.2% and inhibited *CD86* expression by 89.9 ± 12.2%. This inhibitory effect seems to be specific to SAC stimulation because *CD80* and *CD86* upregulation by TD stimulation was not affected (Fig. 2a). Distinct from *CD80* and *CD86*, SAC-induced *CD69* expression was decreased by CTLA-4-Ig in 4 donors but was unchanged in the other 3 donors (Fig. 2a).

To confirm the suppressive effect of CTLA-4-Ig on these activation markers, we conducted immunofluorescent staining. We found that CTLA-4-Ig significantly reduced the surface levels of CD80 and CD86 after SAC stimulation. Conversely, the surface CD69 level was not affected (Fig. 2b), indicating that CTLA-4-Ig specifically suppressed CD80 and CD86 but not CD69 expression. Although TD stimulation-induced *CD80* transcription was not influenced by CTLA-4-Ig (Fig. 2a), the surface level of CD80 was significantly reduced (Fig. 2b). Because the binding of CTLA-4-Ig to CD80 and CD86 impedes their detection by immunofluorescence staining, we conducted acidic elution of bound CTLA-4-Ig before staining. The acidic elution removed the bound CTLA-4-Ig on activated B cells (supplemental Fig. 2A). Using the same procedures to detect the absolute expression, a reduction in SAC-induced CD80 and CD86 was observed in the 100 μg/ml CTLA-4-Ig-treated group compared to that of the controls. In contrast, TD-stimulated CD80 and CD86 levels were not affected by CTLA-4-Ig (Fig. 2c). These results are consistent with the RT-q-PCR data and therefore confirmed that CTLA-4-Ig suppressed SAC-induced CD80 and CD86 expression. The steady-state concentration of abatacept in patient blood was 24 μg/ml (1–66 μg/ml); thus, we examined its efficacy at lower concentrations. We found that 10 and 30 μg/ml CTLA-4-Ig inhibited CD80 and CD86 expression to a similar extent as observed in the 100 μg/ml CTLA-4-Ig-treated group (supplemental Fig. 2B). Taken together, these results demonstrated that CTLA-4-Ig reduces SAC-induced CD80 and CD86 expression and limits the accessibility of surface CD80 on TD-stimulated human B cells in vitro.

### CTLA-4-Ig suppresses SAC-induced TNF-α and IL-6 expression in the B cells

**Fig. 3:**
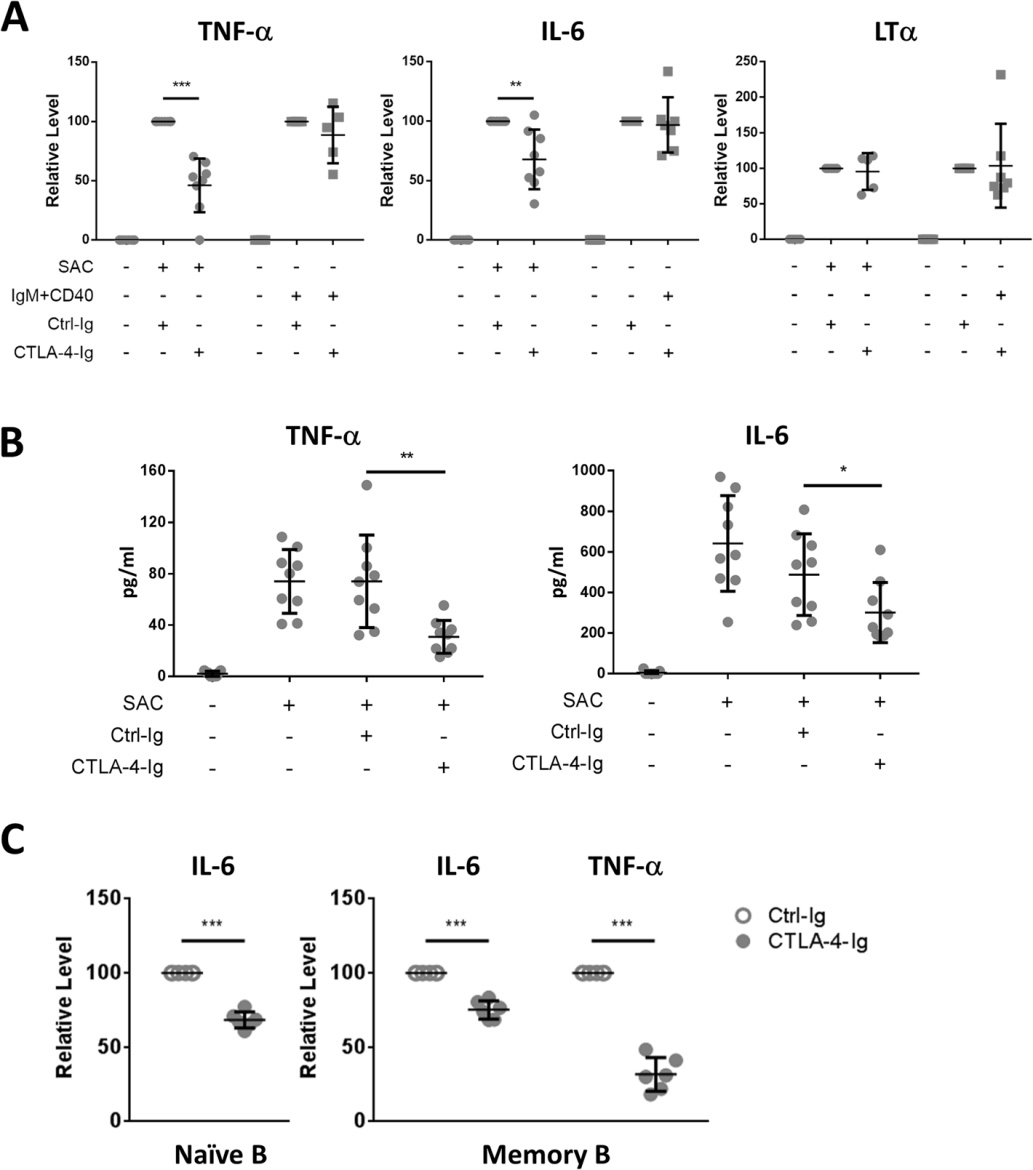
CTLA-4-Ig suppresses SAC-induced TNF-α and IL-6 production in B cell. **a** Purified B cells were stimulated with SAC or anti-IgM plus anti-CD40 antibodies in the presence of CTLA-4-Ig (100 μg/ml) or Ctrl-Ig (100 μg/ml) for 8 h (*TNFA*) or 48 h (*IL6* and *LTA*), and gene expression was measured as described in Fig. 2b. **b** Concentration of TNF-α or IL-6 in the supernatant of the SAC-stimulated B cells was analyzed by ELISA. **c** CTLA-4-Ig suppressed proinflammatory cytokine production by naïve and memory B cells. Sorted naïve and memory B cells were stimulated with SAC in the presence of CTLA-4-Ig or Ctrl-Ig for 8 h (TNFα) or 48 h (IL-6), and the supernatant was collected for ELISA (*n* = 6). The readout for the Ctrl-Ig treatment results was considered 100% for the calculation of the relative level in each donor

Cytokine expression is one of the biological functions of B cells. To further verify the inhibitory effect of CTLA-4-Ig on B cell activation, we stimulated B cells as described above and used RT-q-PCR to measure cytokine expression. The expression of *TNFΑ*, *IL6*, and lymphotoxin-α (*LTA*) was induced by either SAC or TD stimulation. CTLA-4-Ig specifically reduced SAC-induced pro-inflammatory cytokine *TNFA* and *IL6* expression, but it did not affect *LTΑ* expression (Fig. 3a). In contrast, CTLA-4-Ig did not change TD stimulation-induced *TNFΑ*, *IL6*, or *LTΑ* expression (Fig. 3a). On average, *TNFA* expression was suppressed by 53.8 ± 22.7% in all 8 donors and *IL6* was reduced by 37.4 ± 21.7% in 7 of 8 donors (*TNF*, *p* < 0.0001; *IL6*, *p* < 0.01). Consistent with the reduction in mRNA levels, CTLA-4-Ig decreased TNF-α secretion after SAC stimulation by an average of 56.6 ± 8.3% (*p* < 0.001), and IL-6 secretion was reduced by 37.7 ± 12.4% (Fig. 3b, *p* < 0.05). No TNF-α or IL-6 secretion could be detected in the B cells after TD stimulation. Taken together, we concluded that CTLA-4-Ig suppresses SAC-induced TNF-α and IL-6 production but not *LTΑ* expression in human B cells.

Given that CTLA-4-Ig is mainly bound memory B cells (Fig. 1a), we wondered whether CTLA-4-Ig affects memory B cells instead of naïve B cells. To test this hypothesis, we sorted blood naïve and memory B cells and examined their responses to CTLA-4-Ig. The ELISA analysis results revealed that cytokine secretion by both subsets was reduced by CTLA-4-Ig (Fig. 3c). TNF-α was secreted by memory cells but not by naïve B cells after SAC stimulation, and CTLA-4-Ig significantly suppressed TNF-α secretion by the memory B cells. CTLA-4-Ig also lowered IL-6 secretion from both the naïve B cells and memory B cells (Fig. 3c).

**Fig. 4:**
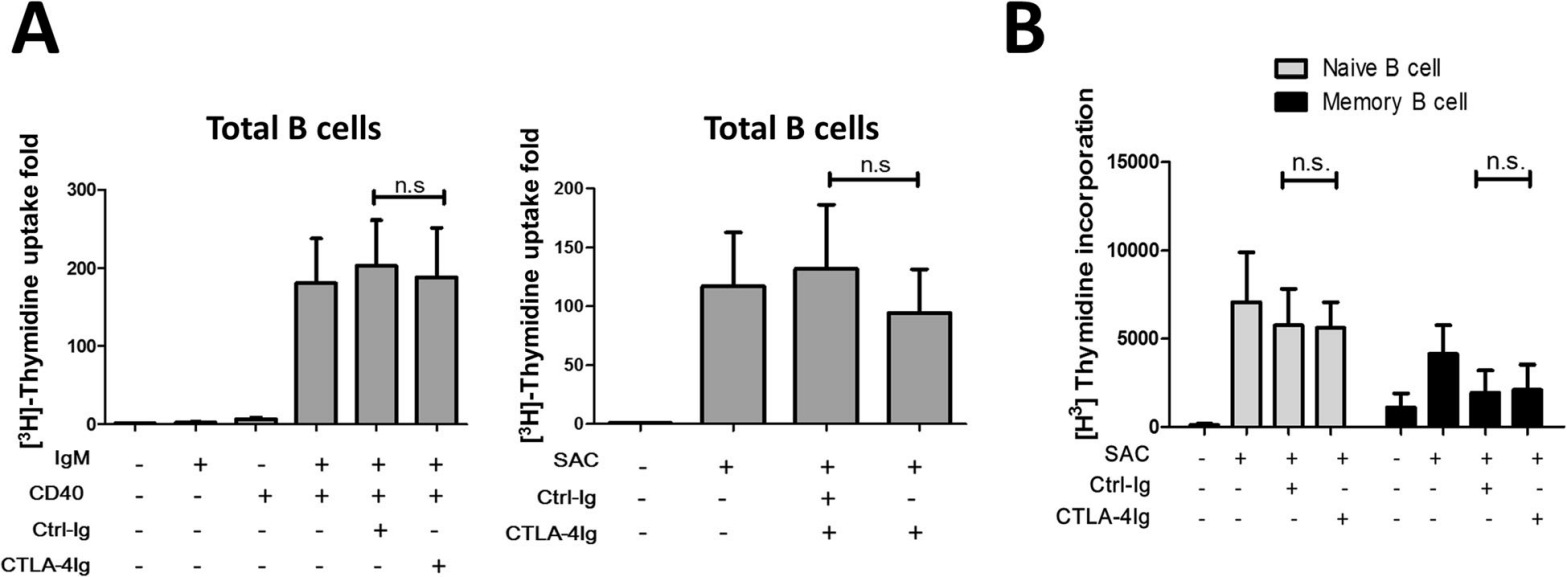
CTLA-4-Ig has no effect on SAC- and TD-induced proliferation of human B cells. **a** CTLA-4-Ig did not affect T-dependent or SAC-induced B cell proliferation. Fifty thousand purified B cells per well were seeded in a 96-well plate and stimulated with SAC or anti-IgM (5 μg/ml) plus anti-CD40 (1 μg/ml) antibodies in the presence of CTLA-4-Ig (100 μg/ml) or Ctrl-Ig (100 μg/ml) for 3 days, and then 1 μCi [^3^H]-thymidine was added to each well and incubated for an additional day before being harvested (*n* = 7). The readout of the medium only group was used to calculate the incorporation (fold) of [^3^H]-thymidine for each donor sample. The bar graph shows the cumulative incorporation (fold) of the samples from 7 donors. **b** CTLA-4-Ig had no effect on naïve or memory B cell proliferation. Sorted naïve and memory B cells were stimulated with SAC in the presence of CTLA-4-Ig or Ctrl-Ig, and [^3^H]-thymidine uptake was measured. The bar graph shows the cumulative incorporation in the samples of 3 donors

Next, we tested the effect of CTLA-4-Ig on B cell proliferation. We found that neither SAC- nor TD-stimulated B cell proliferation was influenced by CTLA-4-Ig, as shown by the ^3^H-thymidine incorporation assay results (Fig. 4a). When the sorted B cells were analyzed, neither naïve nor memory B cell proliferation was found to be influenced by CTLA-4-Ig (Fig. 4b). These results suggest that CTLA-4-Ig has no direct effect on SAC- and TD-induced human B cell proliferation.

### Effect of CTLA-4-Ig on plasma cell differentiation and antibody secretion

**Fig. 5:**
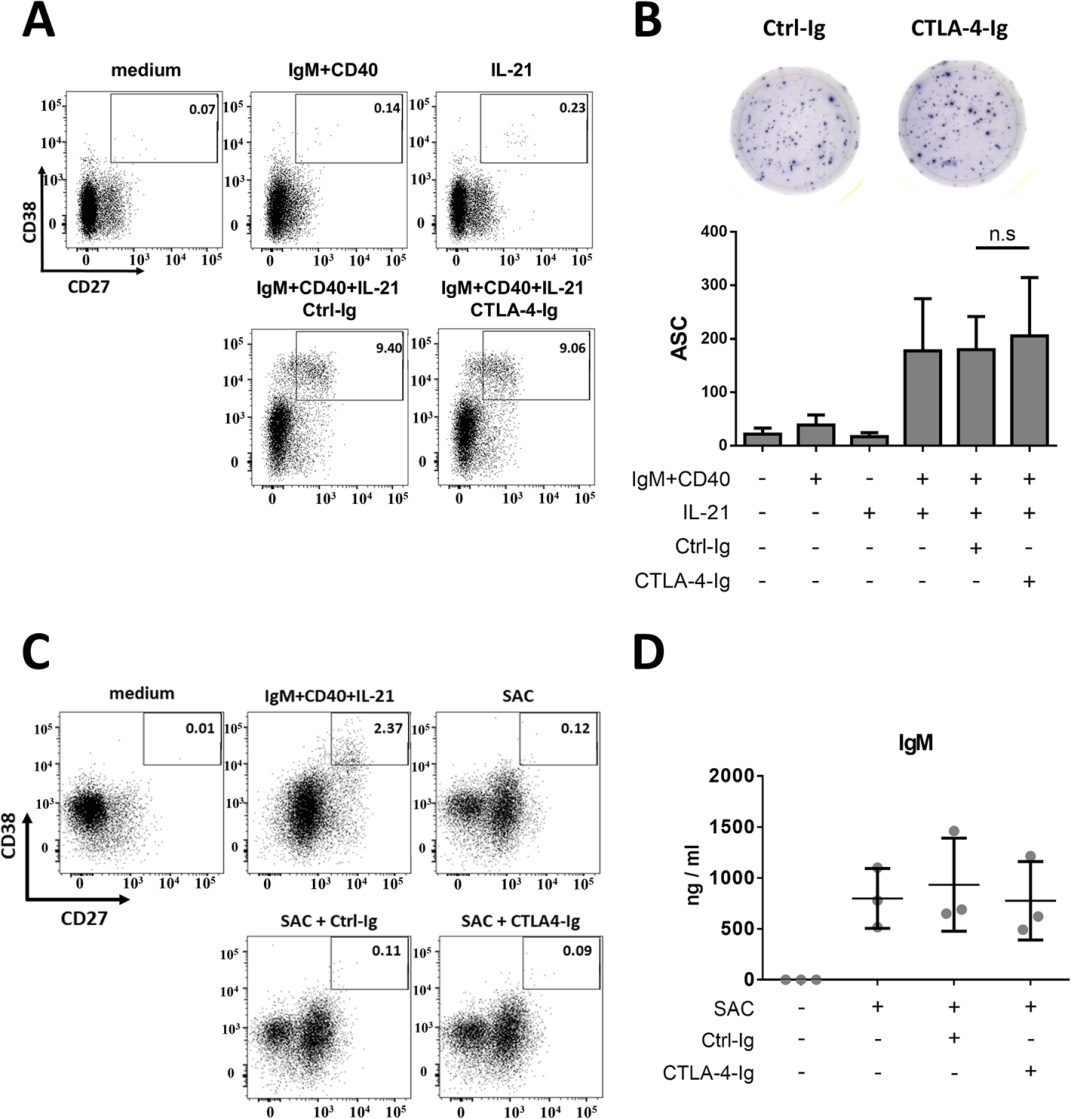
CTLA-4-Ig has no effect on T cell-dependent stimulation- or SAC-induced plasma cell differentiation or antibody secretion. Purified B cells were stimulated with anti-IgM (5 μg/ml), anti-CD40 (1 μg/ml) antibody, and IL-21 (100 ng/ml), or with SAC in the presence of Ctrl-Ig or CTLA-4-Ig for 4 days, and the cells (**a** and **c**) were stained with anti-CD27 and anti-CD38 antibodies to mark plasma cells (CD38^high^CD27^high^; *n* = 3). One representative result is shown. **b** ELISPOT assay results after TD stimulation. Two thousand B cells (activated for 4 days) were seeded on a 96 well plate precoated with anti-hIgG antibody in triplicate, and the cells were incubated 16 h at 37 °C in complete RPMI medium. After removing the cells, anti-hIgG antibody conjugated with biotin was added to mark antibody secreting cells (ASC). The mean spot number was quantified by an AID ELISPOT reader and analyzed by AID ELISPOT software. Upper, one representative result. Lower, cumulative ELISPOT assay results of samples from 3 donors. **d** IgM secretion after SAC stimulation for 3 days. IgM in the supernatant of the activated B cells was measured using ELISA

Previous studies have demonstrated the involvement of CD80 and CD86 on B cells in regulating antibody secretion [25–29]. Therefore we tested whether CTLA-4-Ig directly regulates plasma cell differentiation and/or antibody secretion after human B cell activation. IL-21 is able to induce human plasma cell differentiation under TD simulation in vitro [34]. We stimulated purified B cells with IL-21, anti-CD40, and anti-IgM antibodies to promote plasma cell differentiation and used CD38^high^CD27^high^ as a plasmablast/plasma cell marker. Our data showed that CTLA-4-Ig had no impact on TD-induced plasma cell differentiation (Fig. 5a). To examine the effect of CTLA-4-Ig on antibody secretion, we conducted an ELISPOT assay to prevent the interference of CTLA-4-Ig or Ctrl-Ig when quantifying secretory IgG by ELISA. The analysis showed no significant difference between the Ctrl-Ig- and CTLA-4-Ig-treated B cells in either the frequency of IgG-secreting cells or in the amount of antibody secreted per cell (Fig. 5b and data not shown, respectively). In contrast to TLR7- or TLR9-stimulation, SAC stimulation was unable to induce IgM secretion from human B cells [35]. We found that SAC failed to stimulate plasmablast/plasma cell differentiation, and CTLA-4-Ig did not affect plasma cell differentiation or IgM secretion (Fig. 5c, d). Overall, we found no significant impact of CTLA-4-Ig on TD stimulation- and SAC-induced plasma cell differentiation or antibody secretion.

### CTLA-4-Ig reduces the accessibility of CD80 and CD86 on the memory B cells in vivo

**Fig. 6:**
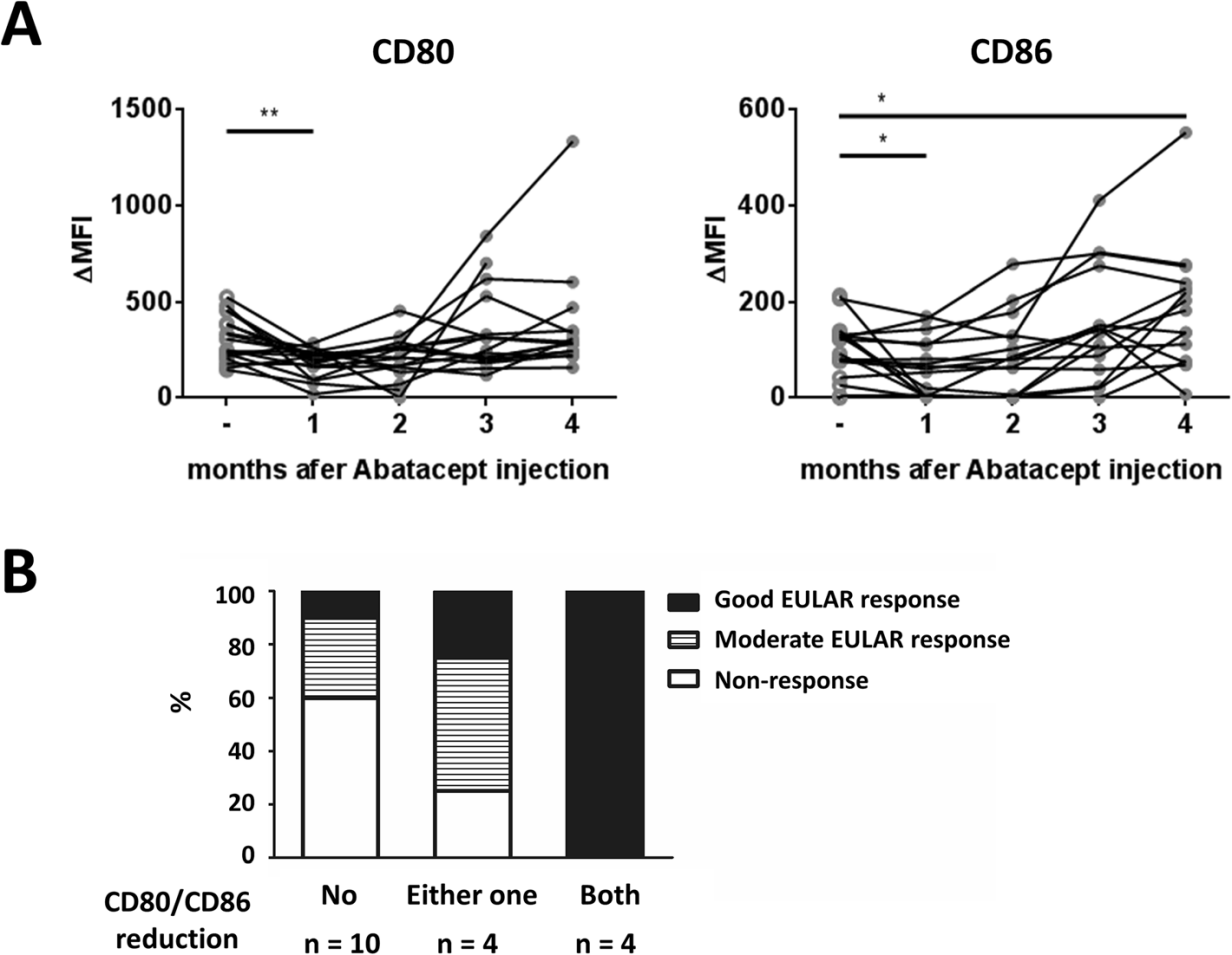
Levels of CD80/CD86 on the surface of the memory B cells correlate with disease severity in the RA patients. **a** Surface level of CD80 or CD86 on peripheral blood memory B cells of RA patients during abatacept treatment was monitored for 4 months (*n* = 18). PBMCs from the RA patients were stained with anti-CD20 and anti-CD27 antibodies in the presence of anti-CD80 or anti-CD86 antibodies. The analysis of CD80 or CD86 level was gated on memory (CD20^+^CD27^+^) cells. The surface level (ΔMFI) was calculated as the mean fluorescence intensity level of CD80/CD86 minus the isotype control. **b** Comparison of the responses of the RA patients with or without decreased CD80 or CD86 levels on the surface of the memory B cells. EULAR, European League Against Rheumatism collaborative initiative classification criteria. The patients were classified as good responders, moderate responders, and non-responders according to the DAS28-ESR levels and their improvement from the baseline 6 months after abatacept treatment

Given that CTLA-4-Ig decreased SAC-induced CD80 and CD86 expression and blocked TD-stimulated CD80 in the in vitro assays, we tested whether CTLA-4-Ig has a similar effect on human B cells in vivo. Blood was drawn from the patients with RA before or each month after receiving abatacept treatment. The characteristics of the enrolled patients are listed in Table 1. Immunofluorescence staining was used to monitor the levels of CD80 and CD86 on the surface of the memory B cells in the blood of the RA patients. Our results showed that the anti-CD80 staining level on the memory B cells was reduced during the first 2 months after abatacept treatment; however, the staining level was restored to baseline or increased starting the 3rd month after the initial treatment (Fig. 6a, *p* < 0.01); it should be noted that these patients continuously received abatacept during the experimental period. Similarly, the anti-CD86 staining level on the surface of the memory B cells in these RA patients was reduced 1 month after the treatment and was gradually restored to baseline or increased starting in the 3rd month after the initial treatment (Fig. 6a, *p* < 0.05). When we performed acid elution to detect the real expression of CD80 and CD86 on the memory B cells of another 3 patients with RA, we observed a combination of different effects, including blocking, reduction, and no influence of abatacept treatment. Acidic elution increased the CD80 levels in all the samples, but it only enhanced the levels of CD86 in some of the samples (Supplemental Fig. 3A and B). The levels of CD80 and CD86 were blocked in the surface of the memory B cells from patient P27. The level of CD80 was not affected, but the level of CD86 was reduced in the memory B cells from patient P28. In the memory B cells from patient P29, the CD80 and CD86 levels were both reduced and blocked 2 and 3 months after the treatment (Supplemental Fig. 3C). On the basis of these observations, our results suggest that continuous treatment with CTLA-4-Ig may lead to a blocking or reduction of CD80 and CD86 on the surface of the memory B cells in the blood of the RA patients for at least 1 to 2 months in vivo*.*

### Surface levels of CD80 and CD86 on memory B cells correlate with clinical outcome in the RA patients

To study the significance of CD80/CD86 blockade in vivo, we analyzed the correlation between CD80/CD86 reduction on memory B cells and disease activity in the RA patients. The patients were classified as good responders, moderate responders, and non-responders according to the DAS28-ESR levels and their improvement with respect to the baseline after 6 months of abatacept treatment. In the third month of abatacept treatment, 4 patients had a decrease in both CD80 and CD86, 4 patients had a decrease in either CD80 or CD86, and 10 patients had no reduction in either CD80 or CD86 on their memory B cells. Interestingly, all 4 patients with a decrease in both CD80 and CD86 had a good EULAR response, 1 of 4 patients with a reduction in either CD80 or CD86 had a good response, but only 1 of 10 patients without a reduction in either CD80 or CD86 had a good response (Fig. 6b). The group of RA patients with a reduction in both CD80 and CD86 had a higher rate of good EULAR responses than those in the group with no reduction in the CD80 or CD86 levels (100.0% vs. 14.3%, *p* = 0.003). These findings imply that CTLA-4-Ig blocking and/or reduction of CD80 and CD86 on the surface of the memory B cells negatively correlates with disease activity and may predict a good clinical response in RA patients.

## Discussion

CTLA-4-Ig is known to suppress T cell activation and regulate dendritic cell functions; however, relatively little is known about its impacts on B cells. Here, we showed that CTLA-4-Ig specifically suppressed the expression of *CD80*, *CD86*, *TNFA*, and *IL6* but not that of *LTA* after human B cells were stimulated with SAC in vitro. Since the transcription levels of *CD80*, *CD86*, *TNFA*, and *IL6* were reduced, it is highly likely that CTLA-4-Ig binding to B cells transduces a signal that initiates a decrease in the expression of these genes. Our data pinpoint the reduction of B7 and proinflammatory cytokine expression in human B cells after SAC stimulation as another means for CTLA-4 to regulate B cell responses.

In this study, we observed that CTLA-4-Ig directly downregulated SAC-induced pro-inflammatory cytokine (TNF-α and IL-6) production in B cells in vitro. CTLA-4-Ig not only reduced surface CD80 and CD86 proteins but also dramatically inhibited SAC-induced *CD80* and *CD86* transcription (Fig. 2a). The inhibition appeared to be specific because the expression of *LTA*, *IDO1*, or *IL10* was not affected (Fig. 3 and data not shown). These observations support our hypothesis that CTLA-4-Ig binds and transduces a signal to regulate B cell activation. In accordance with our findings, abatacept has been reported to interact with cultured synovial macrophages from RA patients and to downregulate TNF-α, IL-6, and IL-1β production when macrophages were cocultured with Jurkat T cells [22]. Kowalczyk et al. demonstrated that CTLA-4-Ig reduces LPS-induced CD80 and CD86 at both the mRNA and protein levels in dendritic cells [36]. In contrast to the induction of IDO-1 activity in dendritic cells, CTLA-4-Ig did not induce *IDO1* expression in human B cells (data not shown). We tried several conditions but failed to detect *IDO1* or *IDO2* expression in the B cells in our experiments (data not shown). It is possible that the B cells may not be able to express IDO under these conditions. Given that CTLA-4-Ig can reduce SAC-induced proinflammatory cytokine production and CD80/CD86 expression in B cells and that it has similar effect on other APCs such as macrophages and dendritic cells, these findings reveal a universal role of CTLA-4-Ig in modulating the functions of all three major APCs.

Our in vitro assays demonstrated that CTLA-4-Ig treatment had no impact on TD- or SAC-induced plasmablast differentiation and antibody secretion (Fig. 5), which seems to be inconsistent with the observations that CTLA-4-Ig reduces antibody responses against either TD or TI antigens in vivo [17–19]. Because activated T cells provide necessary signaling (such as that related to CD40L and ICOS) and secrete IL-21 to induce plasma cell differentiation and class switching, the blocking or reduction of CD80 and CD86 on B cells may explain the discordance between the in vitro and in vivo results. Here, we found that CTLA-4-Ig strongly inhibited SAC-induced CD80 and CD86 expression in the B cells (Fig. 2c). Although SAC can stimulate B cell activation and proliferation without T cell involvement, the upregulation of CD80/CD86 on B cells increases the chance and time of B cells to interact with T cells to promote B cell activation and subsequent plasma cell differentiation and class switching. In our in vitro differentiation system, we provided sufficient, even excess, stimuli to induce B cell activation and differentiation. Without T cell involvement, CTLA-4-Ig had no direct effect on plasmablast differentiation or antibody secretion. Overall, our results imply that the reduction in the humoral response by CTLA-4-Ig in vivo is most likely due to a decrease in T cell activation and to an interference of the T cell-B cell interaction.

Although CTLA-4-Ig did not reduce transcription levels, the surface CD80 was significantly blocked under TD stimulation (Fig. 2b). This result is attributed to CTLA-4-Ig impeding the ability of the anti-CD80 antibody to bind its cognate antigen, as suggested by the acid elution experiment results (Supplemental Fig. 2). However, a recent report demonstrated that abatacept treatment decreased surface CD80/CD86 protein levels on human B cells by dynamin-dependent internalization in vitro [24]. It is possible that both mechanisms are involved in the downregulation of CD80 and CD86 on the surface of human B cells. When we performed acid elution to detect the real expression of surface B7 molecules on the memory B cells from another 3 patients with RA, we found that the abatacept treatment either blocked or reduced CD80 and CD86 expression on the memory B cells in 2 of 3 patients (Supplemental Fig. 3C). Although the sample size was limited, this observation suggests that continuous treatment of CTLA-4-Ig in the RA patients caused a blocking or decrease in CD80 and CD86 on the surface of the memory B cells. We speculate that the true decrease in CD80/CD86 levels is likely due to CTLA-4-Ig-induced internalization as indicated in the in vitro study [24]. More importantly, restoration of CD80/CD86 expression on the memory B cells in a portion of the RA patients after the 3rd month was associated with high disease activity (Fig. 6b), suggesting that the availability of CD80/CD86 on the B cells is critical for the pathogenesis of RA in humans. Our observations are consistent with previous findings showing that B cells are the major APCs for T cell activation [13] and that the expression of CD80/CD86 on the B cells is essential for the autoreactive T cell activation in arthritic mice [14]. Therefore, the blocking or decrease in CD80/CD86 on the surface of the B cells and subsequent suppression of B cell-mediated T cell activation by CTLA-4-Ig may contribute to the alleviation of disease activity in RA patients.

The involvement of CD28, CD80, and CD86 in the humoral response has been demonstrated in animal models. CD28-knockout mice generated lower basal levels of IgG1 and IgG2b and had defective class switching [37]. Conditional deletion of CD80 and CD86 in B cells also reduced anti-influenza IgG antibody production in an infection model in vivo [38]. Despite the valuable insights obtained from these animal studies, whether B7 molecules directly control Ig production is not clear. Using an in vitro system, Jeannin et al. discovered that anti-CD86 mAb potentiates IgE and IgG4 production in tonsillar B cells [29]. Here, we observed no direct effect of CTLA-4-Ig on TD-induced IgG production or on SAC-induced IgM secretion (Fig. 5). Because anti-CD86 mAbs and CTLA-4-Ig may have different affinities for CD86, and because they may transduce distinct signals in B cells, we cannot exclude a possible direct role of B7 molecules in regulating antibody production. However, since mAbs against CD80 and CD86 might not recapitulate the binding of CD28 to its native ligands, using CD28-Ig or a CD28 recombinant protein in a B cell-only culture system may be a better way to further clarify this question.

We observed that CTLA-4-Ig significantly suppressed SAC-induced CD80, CD86, TNF-α, and IL-6 expression in human B cells. The possible mechanism by which CTLA-4-Ig modulates SAC-induced B cell activation is unknown. In dendritic cells, only plate-bound, not soluble CTLA-4-Ig, is able to decrease CD80/CD86 transcription, and CTLA-4-Ig induces STAT3 phosphorylation followed by suppression of NF-κB activation, leading to a reduction in CD80/CD86 gene expression [36]. Other studies have also reported the involvement of NF-κB activation in the upregulation of CD86 in B cells [39] and dendritic cells [40]. Whether CTLA-4-Ig binding to B cells reduces NF-κB activation and stimulates STAT3 activation requires further investigation. Another question we did not address in this study is whether the membrane form of CTLA-4 suppresses the expression of SAC-induced CD80, CD86, TNF-α, and/or IL-6. Stimulation of B cells in the presence of T cells expressing membrane CTLA-4 or with plate-bound CTLA-4-Ig will help to clarify this question.

In Fig. 6a, we found that the expression of CD80 and CD86 on the surface of the memory B cells from the RA patients was significantly blocked 1 month after abatacept treatment, and less blocked overtime. The acidic elution experiments suggested that both blocking and reduction by abatacept were responsible for the reduction in B7 molecules (supplemental Fig. 3C). More studies with a larger sample size of RA patients are required to confirm whether the decrease in B7 molecules is due to internalization and/or reduction. Another interesting observation in Fig. 6a is that the levels of CD80 and CD86 were restored or even increased in a portion of the RA patients 3 months after the treatment. It is possible that the real expression of CD80 and CD86 on the surface of the memory B cells may be increased even after continuous treatment of abatacept for 6 months. We speculated that the chronic inflammatory status in the RA patients may upregulate the expression of CD80/CD86 in the B cells. For example, autoantigens in the RA patients may induce the expression of CD80 and CD86 by binding B cell receptors and TLRs in the B cells. In addition, TNF-α levels are higher in the RA patients and TNF-α is able to increase CD80 and CD86 expression in human B cells [41].

A previous study has suggested that abatacept is more effective in RA patients who are positive for anti-citrullinated protein antibodies (ACPA) [42]. Similarly, a French registry study showed that positivity of baseline ACPA, but not RF, was associated with good EULAR response in RA patients treated with abatacept [43]. In addition, treatment with abatacept can induce the conversion to ACPA/RF seronegative status in RA patients, implying that CTLA-4-Ig may influence B cell functions [44]. Our findings indicated that CTLA-4-Ig binding to CD80/CD86 may modulate antigen-specific B cell functions directly, which may be another mechanism for abatacept to regulate disease activity of RA. In the current study, the EULAR response after 6 months of the abatacept treatment was observed in 11 (61.1%) of the 18 patients (good response, 33.3%; moderate response, 27.8%). The proportion of RF-positivity was similar in the EULAR responders and non-responders (9 of 11, 81.8% vs. 7 of 7, 100.0%, *p* = 0.497). In contrast, the proportion of ACPA-positivity was higher in the EULAR responders in comparison with the non-responders (9 of 11, 81.8% in responders vs. 2 of 7, 28.6% in non-responders, *p* = 0.049). Further studies with a larger sample size are needed to identify whether ACPA-positivity is associated with a better response to abatacept in Taiwan cohort of RA.

## Conclusion

Using an in vitro system, we showed that CTLA-4-Ig directly suppressed SAC-induced CD80, CD86, TNF-α, and IL-6 expression in human B cells. Interestingly, abatacept treatment blocked and/or decreased CD80 and CD86 levels on the surface of the memory B cells of RA patients for 1 or 2 months. The levels of B7 molecules on the surface of memory B cells is associated with the disease activity of RA in vivo, which may be the result of impaired T cell-B cell interactions and subsequent T cell activation. Taken together, our results indicate that CTLA-4-Ig binding to CD80/CD86 may induce signaling in human B cells to suppress SAC-induced B cell activation and that it blocks and/or reduces CD80/CD86 on memory B cells in vivo. Our findings provide another point of view for studying the mechanisms by which CTLA-4-Ig regulates humoral responses.

## Supplementary information

**Additional file 1: Figure S1.** Dose-dependent binding of CTLA-4-Ig on activated human B cells. Purified blood CD19^+^ B cells were stimulated with or without anti-IgM (5 μg/ml) and anti-CD40 (1 μg/ml) antibodies for 3 days. (A) After washing, levels of CD80 and CD86 were examined using immunofluorescent staining and flow cytometry analysis. Medium, untreated B cells; IgM+CD40, activated B cells. Gray peaks, isotype control; black line, anti-CD80/86 antibody. (B) The binding of various concentrations (1, 10, and 100 μg/ml) of CTLA4-Ig (black lines) or L6 control protein (Grey peaks) on untreated and activated human B cells. Data are representative of 2-4 independent experiments. **Figure S2.** The effect of CTLA-4-Ig on the levels of CD80/CD86 on the surface of activated human B cells. (A) CTL4-Ig treatment prevented anti-CD80 antibody binding to TD stimulation-activated B cells. Purified blood CD19^+^ B cells were stimulated with anti-IgM (5 μg/ml) and anti-CD40 (1 μg/ml) antibodies in the presence of 100 μg/ml CTLA-4-Ig or L6-Ig control protein (Ctrl-Ig) for 2 days. The activated cells were split in half. One half of the cells were incubated with acid elution buffer for 4 mins at room temperature (Acid wash) and the other half were left untreated (w/o acid wash). After PBS washing, both parts of the cells were stained with anti-CD80, anti-CD86, and anti-IgG-Fc antibodies. Anti-IgG-Fc antibody was used to detect CTLA-4-Ig bound on the cell surface. Black lines, cells activated in the presence of CTLA-4-Ig; gray peaks, cells activated in the presence of Ctrl-Ig. The numbers in the upper right corner is the percentage of marker positive cells in the Ctrl-Ig treated (gray) or CTLA-4-Ig treated (bold) cells. The peak in the right of the anti-IgG-Fc staining histogram is surface IgG^+^ (class switched memory) B cells. (B) CTLA-4-Ig treatment reduced SAC-induced CD80 and CD86 levels on the surface of the B cells. CD19^+^ B cells were stimulated SAC in the presence of various concentrations (10, 30, or 100 μg/ml) of CTLA-4-Ig or L6-Ig control protein (Ctrl-Ig) for 2 days. After acid wash, the levels of CD80 and CD86 on the CTLA-4-Ig- (black lines) or Ctrl-Ig- (grey peaks) treated cells were examined using immunofluorescent staining. One representative experiment out of 4 was shown. **Figure S3.** The effect of abatacept on the levels of CD80/CD86 on the surface of the memory B cells from 3 patients with RA. The PBMCs isolated from 3 patients with RA were split in half. One half of the cells were incubated with acid elution buffer for 4 mins at room temperature (acid wash) and the other half were left untreated. After PBS washing, both parts of the cells were stained with anti-CD80, anti-CD86, anti-CD27, anti-IgD, anti-CD20, and anti-IgG-Fc antibodies. The label on top of the histogram indicates the time after abatacept injection. (A) The levels of CD80 and CD86 in the memory B cells of one of the 3 RA patients were shown. The analysis of CD80 or CD86 level was gated on memory (CD20^+^CD27^+^) cells. Black lines, the cells treated with acid wash; gray peaks, the cells without acidic elution. (B) The levels of CD80 and CD86 on the surface of the memory B cells in the PBMCs of the 3 RA patients. Gray dots, samples without acidic elution; open circles, samples with acidic elution. (C) The trend of CD80 and CD86 expression on the memory B cells of the same 3 RA patients in B before (top) and after acid wash (bottom).

## Data Availability

Not applicable.

## Abbreviations

CTLA-4: Cytotoxic T lymphocyte-associated antigen-4
DAS28-ESR: Disease Activity Score 28 using ESR
ESR: Erythrocyte sedimentation rate
IDO: Indoleamine 2,3-dioxygenase
IL-6: Interleukin-6
LT-α: Lymphotoxin-α
RA: Rheumatoid arthritis
RF: Rheumatoid factor
SAC: *Staphylococcus aureus* Cowan strain
TNF-α: Tumor necrosis factor-α
VAS ptGA: Patient global assessment on a visual analog scale
