# Supplementary material for: Cytotoxic T lymphocyte-associated antigen-4-Ig (CTLA-4-Ig) suppresses Staphylococcus aureus-induced CD80, CD86, and pro-inflammatory cytokine expression in human B cells
Source: Arthritis Res Ther. 2020 Mar 30;22:64. doi: 10.1186/s13075-020-2138-x (PMC7106629; doi:10.1186/s13075-020-2138-x)
Supplement: Supplementary file 1 — Additional file 1: Figure S1. Dose-dependent binding of CTLA-4-Ig on activated human B cells. Purified blood CD19+ B cells were stimulated with or without anti-IgM (5 μg/ml) and anti-CD40 (1 μg/ml) antibodies for 3 days. (A) After washing, levels of CD80 and CD86 were examined using immunofluorescent staining and flow cytometry analysis. Medium, untreated B cells; IgM+CD40, activated B cells. Gray peaks, isotype control; black line, anti-CD80/86 antibody. (B) The binding of various concentrations (1, 10, and 100 μg/ml) of CTLA4-Ig (black lines) or L6 control protein (Grey peaks) on untreated and activated human B cells. Data are representative of 2-4 independent experiments. Figure S2. The effect of CTLA-4-Ig on the levels of CD80/CD86 on the surface of activated human B cells. (A) CTL4-Ig treatment prevented anti-CD80 antibody binding to TD stimulation-activated B cells. Purified blood CD19+ B cells were stimulated with anti-IgM (5 μg/ml) and anti-CD40 (1 μg/ml) antibodies in the presence of 100 μg/ml CTLA-4-Ig or L6-Ig control protein (Ctrl-Ig) for 2 days. The activated cells were split in half. One half of the cells were incubated with acid elution buffer for 4 mins at room temperature (Acid wash) and the other half were left untreated (w/o acid wash). After PBS washing, both parts of the cells were stained with anti-CD80, anti-CD86, and anti-IgG-Fc antibodies. Anti-IgG-Fc antibody was used to detect CTLA-4-Ig bound on the cell surface. Black lines, cells activated in the presence of CTLA-4-Ig; gray peaks, cells activated in the presence of Ctrl-Ig. The numbers in the upper right corner is the percentage of marker positive cells in the Ctrl-Ig treated (gray) or CTLA-4-Ig treated (bold) cells. The peak in the right of the anti-IgG-Fc staining histogram is surface IgG+ (class switched memory) B cells. (B) CTLA-4-Ig treatment reduced SAC-induced CD80 and CD86 levels on the surface of the B cells. CD19+ B cells were stimulated SAC in the presence of various concentra [file 13075_2020_2138_MOESM1_ESM.docx]

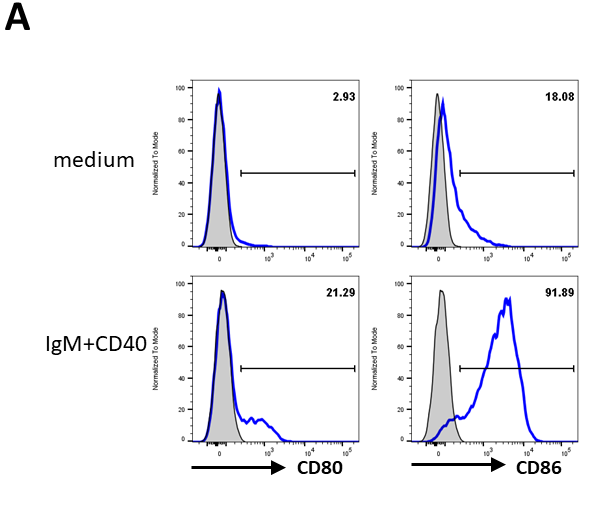

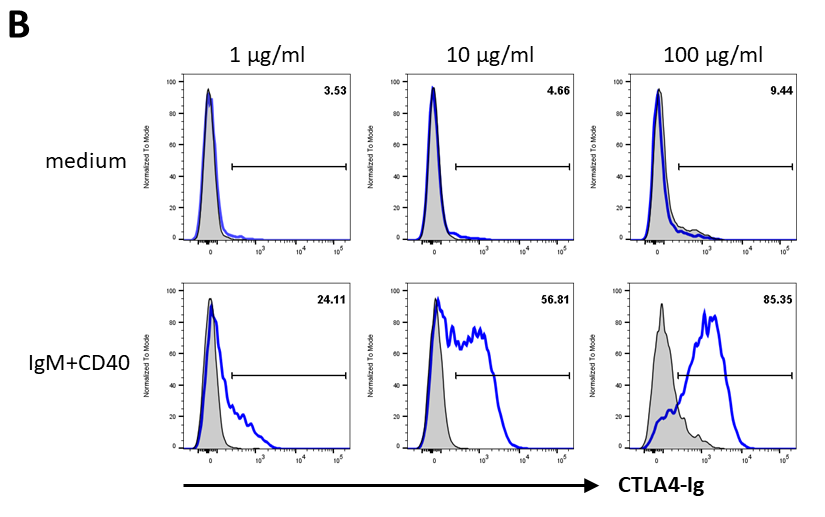


**Figure 1. Dose-dependent binding of CTLA-4-Ig on activated human B cells**

Purified blood CD19^+^ B cells were stimulated with or without anti-IgM (5 μg/ml) and anti-CD40 (1 μg/ml) antibodies for 3 days. (A) After washing, levels of CD80 and CD86 were examined using immunofluorescent staining and flow cytometry analysis. Medium, untreated B cells; IgM+CD40, activated B cells. Gray peaks, isotype control; black line, anti-CD80/86 antibody. (B) The binding of various concentrations (1, 10, and 100 μg/ml) of CTLA4-Ig (black lines) or L6 control protein (Grey peaks) on untreated and activated human B cells. Data are representative of 2-4 independent experiments.

Supplemental Figure 2


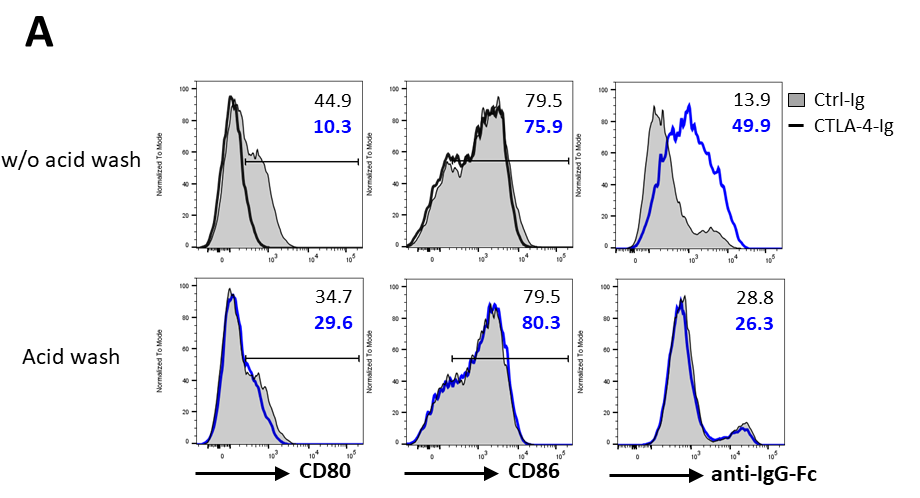

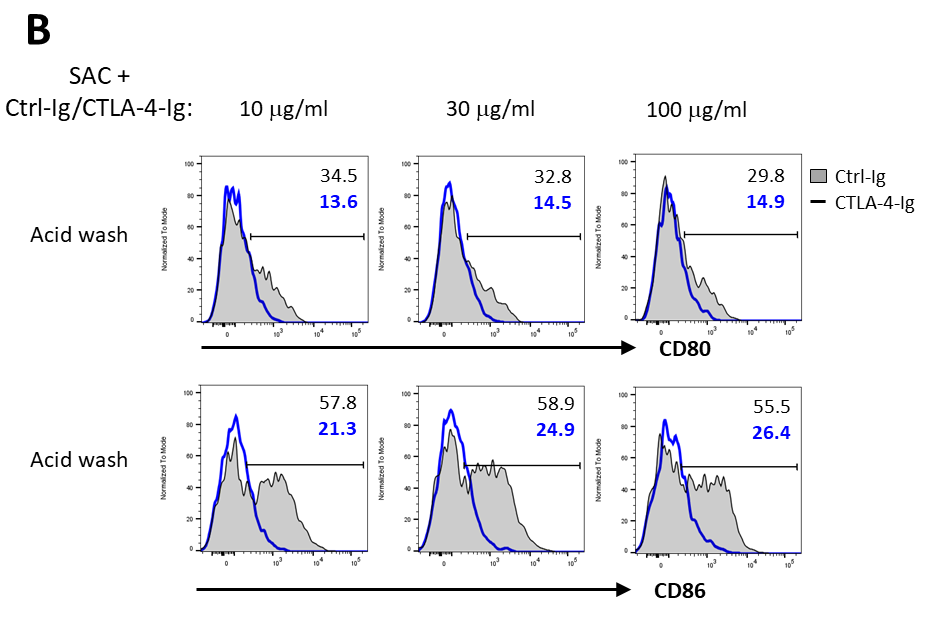


**Figure 2. The effect of CTLA-4-Ig on the levels of CD80/CD86 on the surface of activated human B cells.**

1. CTL4-Ig treatment prevented anti-CD80 antibody binding to TD stimulation-activated B cells. Purified blood CD19^+^ B cells were stimulated with anti-IgM (5 μg/ml) and anti-CD40 (1 μg/ml) antibodies in the presence of 100 μg/ml CTLA-4-Ig or L6-Ig control protein (Ctrl-Ig) for 2 days. The activated cells were split in half. One half of the cells were incubated with acid elution buffer for 4 mins at room temperature (Acid wash) and the other half were left untreated (w/o acid wash). After PBS washing, both parts of the cells were stained with anti-CD80, anti-CD86, and anti-IgG-Fc antibodies. Anti-IgG-Fc antibody was used to detect CTLA-4-Ig bound on the cell surface. Black lines, cells activated in the presence of CTLA-4-Ig; gray peaks, cells activated in the presence of Ctrl-Ig. The numbers in the upper right corner is the percentage of marker positive cells in the Ctrl-Ig treated (gray) or CTLA-4-Ig treated (bold) cells. The peak in the right of the anti-IgG-Fc staining histogram is surface IgG^+^ (class switched memory) B cells.
2. CTLA-4-Ig treatment reduced SAC-induced CD80 and CD86 levels on the surface of the B cells. CD19^+^ B cells were stimulated SAC in the presence of various concentrations (10, 30, or 100 μg/ml) of CTLA-4-Ig or L6-Ig control protein (Ctrl-Ig) for 2 days. After acid wash, the levels of CD80 and CD86 on the CTLA-4-Ig- (black lines) or Ctrl-Ig- (grey peaks) treated cells were examined using immunofluorescent staining. One representative experiment out of 4 was shown.

Supplemental Figure 3


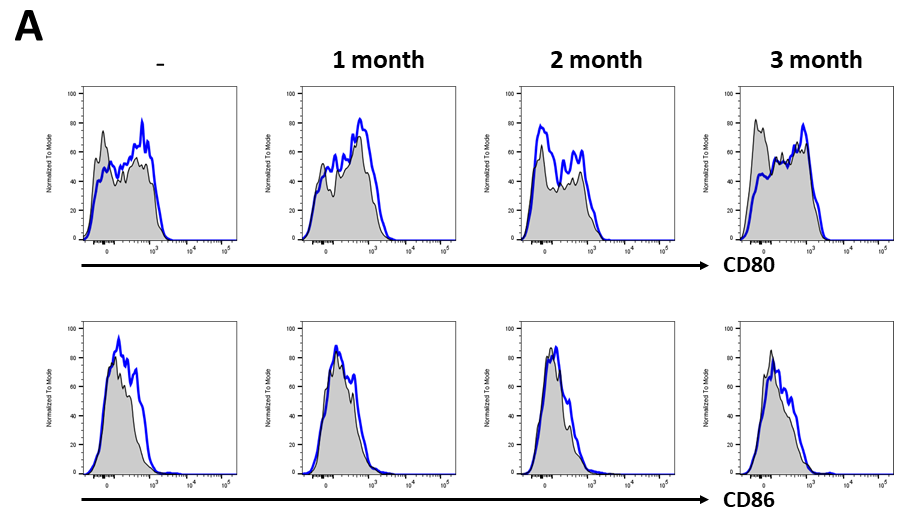


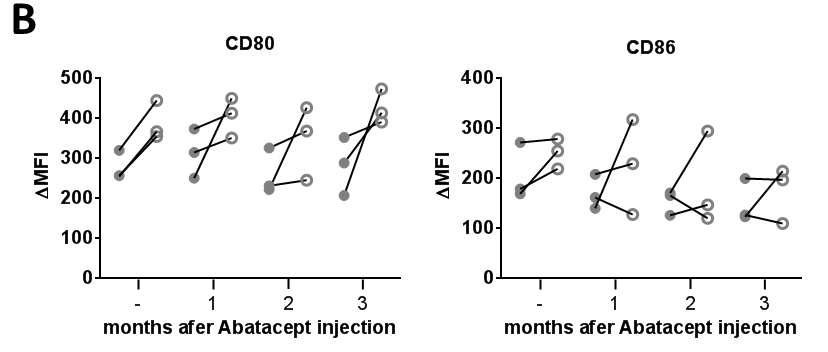


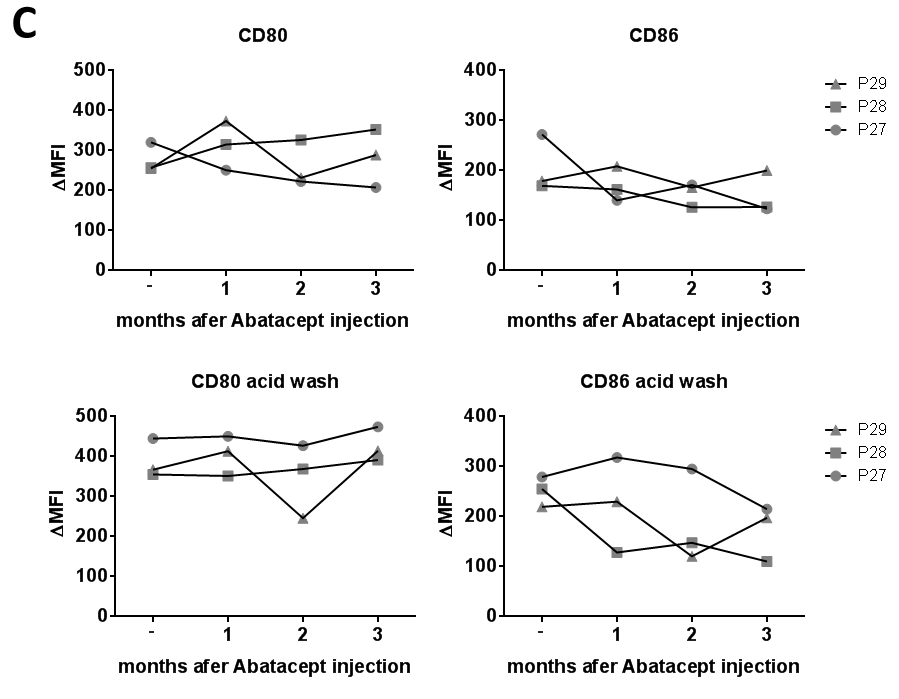


**Figure 3. The effect of abatacept on the levels of CD80/CD86 on the surface of the memory B cells from 3 patients with RA.**

The PBMCs isolated from 3 patients with RA were split in half. One half of the cells were incubated with acid elution buffer for 4 mins at room temperature (acid wash) and the other half were left untreated. After PBS washing, both parts of the cells were stained with anti-CD80, anti-CD86, anti-CD27, anti-IgD, anti-CD20, and anti-IgG-Fc antibodies. The label on top of the histogram indicates the time after abatacept injection. (A) The levels of CD80 and CD86 in the memory B cells of one of the 3 RA patients were shown. The analysis of CD80 or CD86 level was gated on memory (CD20^+^CD27^+^) cells. Black lines, the cells treated with acid wash; gray peaks, the cells without acidic elution. (B) The levels of CD80 and CD86 on the surface of the memory B cells in the PBMCs of the 3 RA patients. Gray dots, samples without acidic elution; open circles, samples with acidic elution. (C) The trend of CD80 and CD86 expression on the memory B cells of the same 3 RA patients in B before (top) and after acid wash (bottom).
